# Confined Ionic Environments Tailoring the Reactivity of Molecules in the Micropores of BEA-Type Zeolite

**DOI:** 10.1021/jacs.4c03405

**Published:** 2024-06-18

**Authors:** Sungmin Kim, Feng Chen, Donald M. Camaioni, Miroslaw A. Derewinski, Oliver Y. Gutiérrez, Yue Liu, Johannes A. Lercher

**Affiliations:** † Institute for Integrated Catalysis and Physical Science Division, Pacific Northwest National Laboratory, Richland, Washington 99354, United States; ‡ Department of Chemistry and Catalysis Research Institute, TU München, Lichtenbergstrasse 4, Garching 85748, Germany

## Abstract

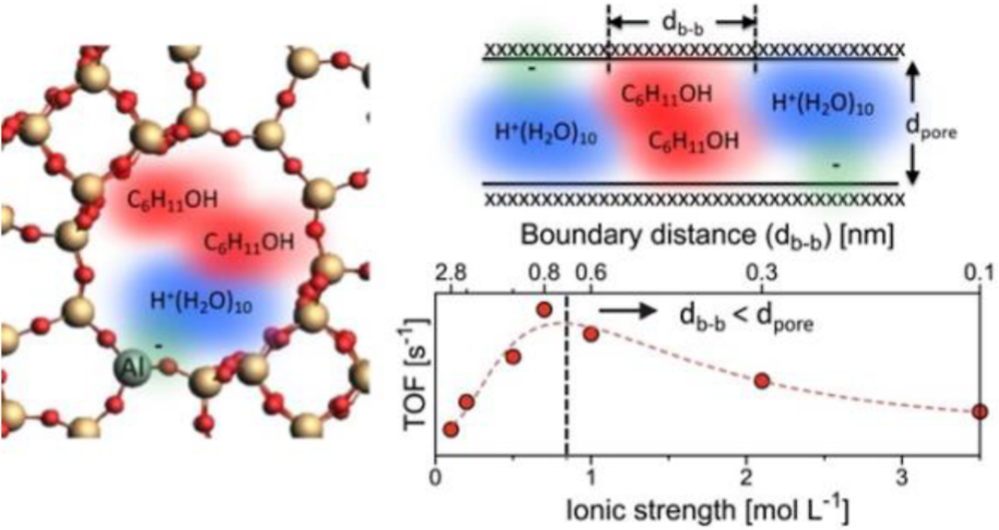

In the presence of water, hydronium ions formed within the micropores of zeolite H-BEA significantly influence the surrounding environment and the reactivity of organic substrates. The positive charge of these ions, coupled with the zeolite’s negatively charged framework, results in an ionic environment that causes a strongly nonideal solvation behavior of cyclohexanol. This leads to a significantly higher excess chemical potential in the initial state and stabilizes at the same time the charged transition state in the dehydration of cyclohexanol. As a result, the free-energy barrier of the reaction is lowered, leading to a marked increase in the reaction rates. Nonetheless, there is a limit to the reaction rate enhancement by the hydronium ion concentration. Experiments conducted with low concentrations of reactants show that beyond an optimal concentration, the required spatial rearrangement between hydronium ions and cyclohexanols inhibits further increases in the reaction rate, leading to a peak in the intrinsic activity of hydronium ions. The quantification of excess chemical potential in both initial and transition states for zeolites H-BEA, along with findings from HMFI, provides a basis to generalize and predict rates for hydronium-ion-catalyzed dehydration reactions in Brønsted zeolites.

## Introduction

Zeolites are crystalline microporous aluminosilicates with well-defined Brønsted acid sites (BAS) and Lewis acid sites (LAS), which result from the substitution of Si^4+^ by Al^3+^ at tetrahedral positions in the framework.^1^ Hence, zeolites are widely used in the chemical industry for sorption, separation, and catalysis.^2−8^ For catalysis, acid sites within zeolite pores have enhanced reactivities compared to those in open environments.^9−12^ This has been attributed to the confinements, stabilizing transition states.^5,6,13−15^ The positive effects for adsorption and stabilization of intermediates have been extensively studied in gas–solid interfaces.^9,10,16,17^ Understanding and controlling the molecular environment of zeolite micropores interacting with reacting molecules in liquid solvents are, however, a formidable challenge.

The environment in the micropores of acidic zeolites is determined by interactions with substrates involving hydrogen bonding or protonation or interactions with surface functionalities.^18,19^ In this complex medium, the organization of the solvent and the reacting molecules greatly influences the nature of active sites in nanoscopic confinements. Thus, understanding the influence of pore environments and molecular structure on the organization of substrates and kinetic parameters is crucial for advancing catalyst design and discovery.

Water in zeolite micropores forms hydronium ions, which induces a lower standard free energy barrier for cyclohexanol dehydration, leading to 2 orders of magnitude higher reaction rates compared to an unconfined aqueous acid solution.^19−21^ The underlying deviation of the ground and transition states from ideal state can be measured by the excess chemical potential.

In our earlier work with HMFI, we found that hydronium ions create a high local ionic strength.^22^ We observed a volcano-like pattern in the turnover frequency (TOF) for the dehydration of cyclic alcohols in the presence of water,^23^ where the maximum of TOF was independent of the substitution of alcohols or the dehydration mechanism, whether through an E1 (sequential C–H and C–O bond cleavage) or an E2 (simultaneous C–H and C–O bond cleavage) mechanism.^19^ To deepen our understanding of the ionic environment within zeolite micropores, it is essential to further explore how hydronium ions and reacting molecules are locally organized, particularly considering the steric constraints, such as the pore diameter of the micropores.

In this work, we aim to fundamentally understand the molecular environment of HBEA micropores that control cyclohexanol dehydration in the presence of water. For this purpose, we used a broad range of Brønsted acid site (BAS) concentrations, minimizing the presence of defect sites by regulating the crystallization rates through the introduction of fluoride ions during the synthesis process. This allows us to characterize the molecular environment of H-BEA micropores and its impact on sorption and catalysis, specifically, the dehydration of cyclohexanol in water. The comparison with the results of dehydration in H-MFI allows a first step toward a generalization of the impact of the hydronium density in micropores.

## Results and Discussion

### Kinetics of HBEA-Catalyzed Cyclohexanol Dehydration in Water

The aqueous-phase dehydration of cyclohexanol was carried out using a series of Beta-type zeolites with varying BAS concentrations, named HBEA with Si/Al ratio (15–400), at 150–180 °C. The physicochemical properties of H-BEA zeolites are tabulated in Table 1.

**Table 1:** Physicochemical Properties of HBEA Zeolites Including the Concentration of BAS and the Volume of Micropores (*V*_micropore_)

zeolite^a^	BAS [mmol g^–^^1^]^b^	*V*_micropore_ [cm^3^ g^–^^1^]^c^	*V*_micropore_ per unit cell [nm^3^]	H^+^(H_2_O)_10_ per unit cell^d^	ionic strength [mol L^–^^1^]^e^
HBEA15	0.820	0.235	1.50	3.16	3.5
HBEA25	0.437	0.213	1.36	1.68	2.1
HBEA50	0.207	0.201	1.28	0.80	1.0
HBEA75	0.163	0.203	1.29	0.63	0.8
HBEA100	0.095	0.194	1.24	0.37	0.5
HBEA200	0.047	0.192	1.22	0.18	0.2
HBEA400	0.020	0.188	1.20	0.08	0.1

a The number represents the Si/Al ratio of the HBEA zeolite.

b The BAS concentrations were quantified by the IR spectra of adsorbed pyridine at 150 °C, using the molar extinction coefficients (0.73 cm/μmol) for the peak area (1565–1515 cm^–1^) normalized by the disc weight.

c The pore volume in micropores was determined from N_2_ physisorption using the *t*-plot method.

d The hydronium ion concentration per unit cell of HBEA was calculated by multiplying the BAS concentration and the weight of unit cell (3840 g mol^–1^), where the composition of the HBEA unit cell is H_*x*_Al_*x*_Si_64–*x*_O_128_.^24^

e Ionic strength is estimated by the normalized BAS concentration, corresponding to the hydronium ion concentration, to the pore volume of the HBEA micropore.

**Figure 1:**
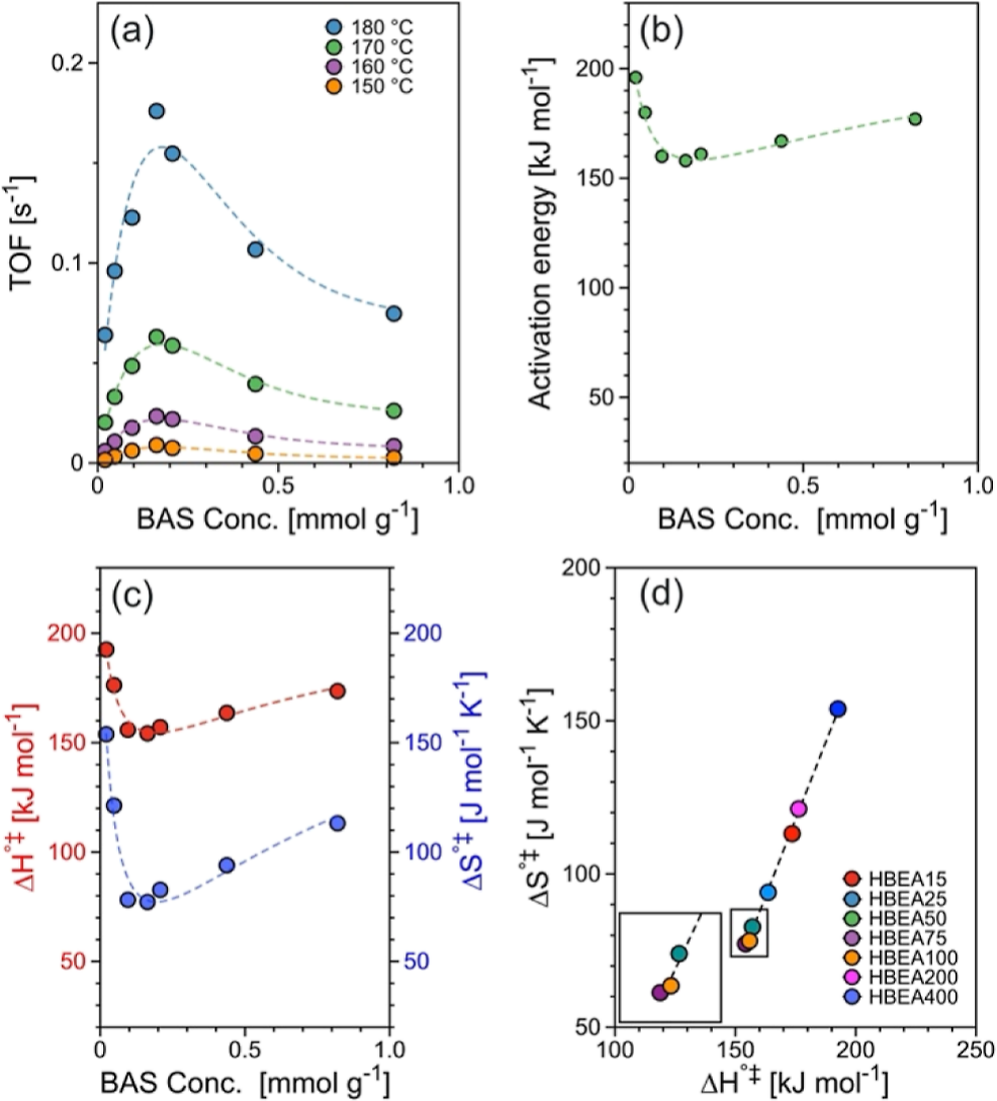
Reaction rate and activation parameters: (a) normalized reaction rate of aqueous-phase dehydration of cyclohexanol (0.3 M cyclohexanol) to BAS at 150–180 °C, (b) activation energy, and (c) activation enthalpy (Δ*H*°^⧧^) and activation entropy (Δ*S*°^⧧^) for aqueous-phase dehydration of cyclohexanol as a function of BAS concentration. (d) Correlation of activation enthalpy (Δ*H*°^⧧^) and activation entropy (Δ*S*°^⧧^) for aqueous-phase dehydration of cyclohexanol on HBEA (Si/Al = 15–400). Initial rates were evaluated at 2–20% of cyclohexanol conversion under zero-order reaction regime, i.e., independent of the cyclohexanol concentration (Figure S1a) and not affected by diffusion limitations (Figure S1b).

Figure S2a shows the reaction rates for cyclohexene formation, while Figure 1a shows the rates normalized to the BAS concentration (i.e., turnover frequency, TOF) as a function of BAS concentration. The TOFs follow a volcano-like trend with changing BAS concentration. Independent of the reaction temperature, maximum activity was observed at 0.16 mmol/g_HBEA_. For instance, the TOF at 150 °C increased almost 6-fold (from 1.6 × 10^–3^ to 9.0 × 10^–3^ s^–1^) with the BAS concentration increasing from 0.02 mmol/g_HBEA_ to 0.16 mmol/g_HBEA_ and decreased to 2.7 × 10^–3^ s^–1^ at 0.82 mmol/g_HBEA_. TOFs increased by up to 2 orders of magnitude with temperature (e.g., from 1.6 × 10^–3^ s^–1^ at 150 °C to 1.8 × 10^–1^ s^–1^ at 180 °C). A similar volcano-type correlation between TOF and BAS concentration was observed before for cyclohexanol dehydration with HMFI.^25^ It is important to highlight that this trend diverges from that seen in gas-phase reactions, such as *n*-pentane cracking and 1-propanol dehydration,^9,19,24^ where the TOF is invariant with BAS concentration owing to the constant strength of BAS.^26^

The apparent activation barrier for aqueous-phase dehydration of cyclohexanol on HBEA (Figures 1b and S2b) showed an inverse-volcano trend as a function of BAS concentration; i.e., it decreased from 177 to 158 kJ mol^–1^, and then increased to 196 kJ mol^–1^. As the reactions were performed in the zero-order regime, the apparent activation energy represents the energy difference between the initial state (cyclohexanol associated with the hydronium ion on the zeolite) and the transition state of the rate-determining step of the E1 or E2 elimination pathway. Mechanistic studies showed that the transition state is associated with the C–H bond cleavage for the E1 path or the concerted C–O/C–H bond cleavage for the E2 path.^27^

The trends of the standard activation enthalpy (Δ*H*°^⧧^) and entropy (Δ*S*°^⧧^) were similar to that of the apparent activation energy (Figures 1c and S2c), i.e., an inverse-volcano correlation with BAS concentration. In turn, the activation enthalpy and entropy were linearly correlated (Figure 1d), indicating a compensation effect, i.e., lower activation enthalpies compensate for lower activation entropy.^9,21^

The presence of charged hydronium ions in the micropores dictates the local arrangement of both hydronium ions and cyclohexanol. During the elimination reaction, a positively charged carbenium ion forms, which is stabilized by the ionic environment. Additionally, the constrained environment in the micropores of zeolites also stabilizes the transition state by van der Waals contacts with the zeolite pore walls. This dual stabilization leads to a reduction in the reaction’s standard free energy barrier, consequently resulting in a substantial increase in the reaction rate.

### Molecular Environment with Hydronium and Its Impact on Catalytic Activity

**Figure 2:**
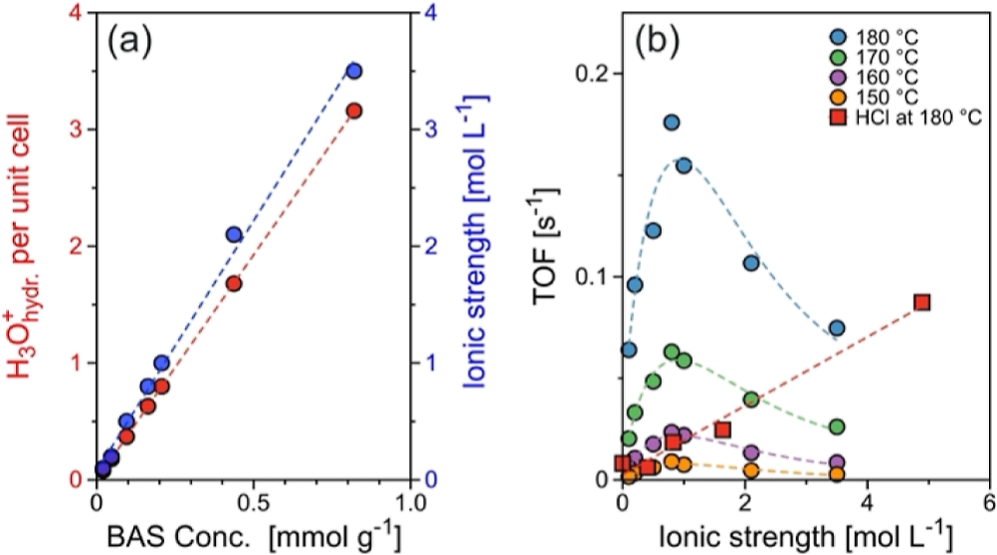
Local hydronium ion concentration and ionic strength: (a) unit cell normalized hydronium ion concentration (H_3_O_hydr_.^+^) and ionic strength as a function of BAS concentration. (b) Reaction rate of cyclohexanol dehydration at 150–180 °C as a function of ionic strength.

In the gas phase, BAS are covalent hydroxyl groups on aluminum-containing tetrahedral positions in the zeolite framework. Such polar groups have a negligible volume in the micropore. In contrast, in the presence of water, the hydronium ion cluster forms a fluxional but sizable species in the pores.^28,29^ Based on adsorption measurements (Figure S3a), we estimated the volume within the micropores of HBEA that is inaccessible to cyclohexanol, effectively representing the volume occupied by the hydronium ions as described in Derivation S2. According to these measurements, the average size of each hydronium ion in the micropore contains 10 water molecules, i.e., H^+^(H_2_O)_10_, which is larger than the hydronium ion clusters in HMFI containing only 8 water molecules (Figure S3b). The ability to form a larger hydrated hydronium ion is ascribed to the lower entropy loss for water adsorption in HBEA compared to that in HMFI.^18,30−32^ The adsorption enthalpies of water on BAS in HBEA and HMFI are comparable, i.e., −66 and −67 kJ mol^–1^ for the first water, and −75 and −77 kJ mol^–1^ for the second water due to hydrogen bonding with BAS and protonation to the bimolecular water cluster.^18,32^ The adsorption enthalpies and entropies consistently changed with the size of the hydration shell for HBEA and HMFI.^32^ This indicates that the proton affinity to water molecules is identical in HBEA and HMFI pores regardless of the size of the hydronium ion clusters. The local concentration of H_3_O_hydr_.^+^ in HBEA micropore was then estimated by normalizing BAS to the volume of the micropore, showing the high local concentration of 0.1–3.5 mol L^–1^. in the HBEA micropore (Table 1 and Figure 2a).

We propose a model that extends the concept of ionic strength (*I*) from homogeneous electrolytes to zeolite micropores. Here, the presence of hydronium ions and the negatively charged framework create an ionic “quasi-solid electrolyte” environment influencing the thermodynamic state of the reacting molecules. In the same manner as for homogeneous electrolytes, the intracrystalline ionic strength (*I*) is determined as a function of the concentration (*c*_*i*_) for the charged species (*z*_*i*_) as follows
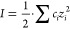
1

The model suggests that the ionic strength influences the intrinsic thermodynamic state of the sorbed substrate, accounted for in the concentration term by the activity coefficient (γ). We use the excess chemical potential (μ^excess^) to describe the impact of the solvent on the solute, i.e., a solute may be stabilized by the solvent (γ < 1, μ^excess^ < 0) or be destabilized (γ > 1, μ^excess^ > 0). Higher ionic strength increases the excess chemical potential of the dissolved substrates and leads to values of γ > 1. As a consequence of the formation of hydronium ions, the ionic strength in HBEA pores increases linearly with the aluminum concentration in the lattice and hence BAS (Table 1 and Figure 2a).

In order to assess the influence of the nonideality induced by ionic strength on the catalytic reaction rate, we define first TOF at a very low concentration of acid sites, infinite dilution of hydronium ions, and then TOF_(ideal)_. This is determined by free-energy barrier under the ideal conditions (Δ*G*_ideal_°^⧧^), which can be expressed by applying the transition-state theory as follows
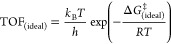
2where *k*_B_, *T*, *h*, and *R* are the Boltzmann constant, temperature, Plank constant, and ideal gas constant, respectively.

Then, the TOF in an ionic environment, i.e., TOF(*I*), can be described as a function of the activity coefficient of the initial state, γ_IS_(*I*), and transitions state, γ_TS_(*I*) (Derivation S2 in the Supporting Information)
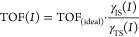
3

Figure 2b shows the rate of cyclohexanol dehydration on HBEA in the presence of water as a function of ionic strength. Owing to the linear relation between ionic strength and BAS concentration, TOFs at 150–180 °C consistently showed a volcano-like trend as a function of ionic strength with the maximum at −1 mol L^–1^. As a reference, the figure also shows TOFs measured during the reaction catalyzed by HCl in a homogeneous solution. In this case, TOF at 180 °C linearly increased from 0.01 s^–1^ at very low ionic strength to 0.08 s^–1^ at 5 mol L^–1^ of ionic strength, in line with the expected positive impact of an increasing ionic strength on the reaction rate. At 180 °C, TOFs were an order of magnitude higher on HBEA (0.18 s^–1^) compared to HCl (0.018 s^–1^) at 0.8 mol L^–1^ ionic strength. This difference is ascribed to the enhanced catalytic activity in the confined space. However, as the ionic strength increases further, the TOF on HBEA decreases to values comparable to those obtained with HCl, i.e., 0.075 and 0.062 s^–1^ (interpolated) at 3.5 mol L^–1^ ionic strength. In analogy to the results with HMFI, we hypothesize that the decrease in rate is caused by the required reorganization of hydronium ions and the substrate in the pores at higher ionic strengths, which leads to a more pronounced charge separation between the hydronium ions and the negatively charged aluminum tetrahedra in the zeolite framework.

### Ionic Strength in the HBEA Micropore Influences the Excess Chemical Potentials

In this section, the impact of the charged environment on the catalytic rates is discussed. For this, we use the component of the excess chemical potential, which is introduced by the concentration of charges (ionic strength), μ_charge_^excess^, that is defined as μ_TS_^excess^ = *RTK*_S_*I*, where *K*_S_ denotes the Setschenow constant, and *I* is the ionic strength in the zeolite pores.

Cyclohexanol dehydration in water is catalyzed by hydronium ions through protonation of the hydroxyl group of cyclohexanol, followed by C–O bond cleavage forming a cyclohexyl carbenium ion and by deprotonation to yield cyclohexene.^9,10^ The standard free-energy barrier is the difference between the transition state, deprotonation of the cyclohexyl carbenium ion by water, and the initial state, cyclohexanol associated with the hydronium ion. In the initial state, the charge-neutral cyclohexanol molecule is destabilized by the ionic strength (μ_IS_^excess^ > 0), thus leading to a proportional increase in the excess chemical potential as follows
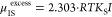
4where *K*_S_ represents the Setschenow constant, which is determined by the adsorption constant and the concentration of hydronium ions (Figure S3c)
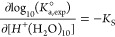
5

On the other hand, the transition state composed of cyclohexyl carbenium ions as a cationic species is stabilized (μ_TS_^excess^ < 0) in the presence of ionic strength, which can be expressed by the extended Debye–Hückel equation
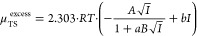
6where *a* is the ion diameter and *A*, *B*, and *b* are constants.

**Figure 3:**
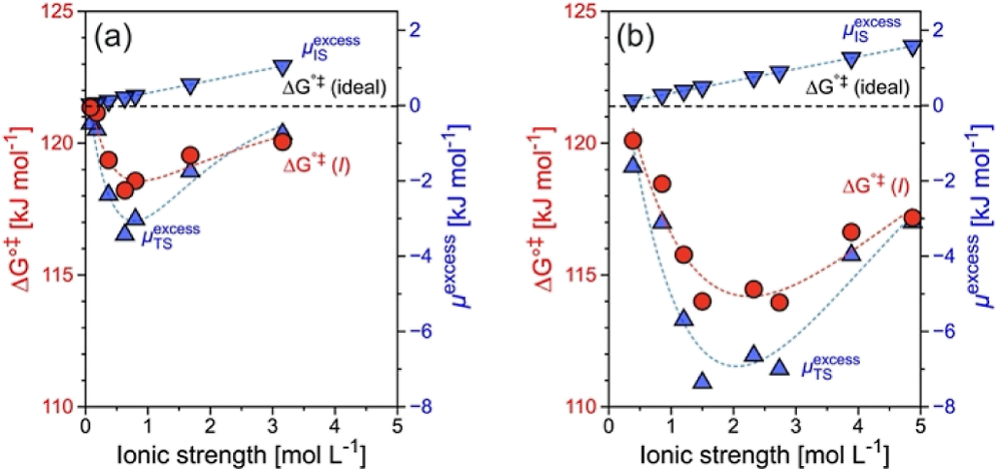
Impact of ionic strength on excess chemical potential: reaction free-energy barriers and excess chemical potential of the initial state (IS) and transition state (TS) under the ideal conditions and ionic strength-induced nonideal conditions in (a) HMFI and (b) HBEA. (a) Reproduced with permission from ref (19). Copyright 2021, AAAS.

The standard free-energy barrier, Δ*G*°^⧧^(*I*), and the excess chemical potential in the initial state (μ_IS_^excess^) and transition state (μ_TS_^excess^) were estimated as described in Derivation S2 in the Supporting Information. The resulting values for HMFI and HBEA are plotted as a function of the ionic strength in Figure 3. The combination of the positive μ_IS_^excess^ and the negative μ_TS_^excess^ leads to lower energy barriers than in the absence of a charged environment, i.e., Δ*G*°^⧧^(ideal) > Δ*G*°^⧧^(*I*). The change of μ_TS_^excess^ is more significant than the change of μ_IS_^excess^. The stabilization of the transition state strongly influences the lower standard free-energy barrier in ionic environments. It is interesting to note that the changes of μ_IS_^excess^ for HMFI and HBEA along with the existing ionic strengths are comparable. This suggests that the excess chemical potential caused by charged species is, as a first approximation, not dependent on pore size and the size of hydronium ion clusters. However, the van der Waals forces within the narrower HMFI pores offer better stabilization of the carbenium-ion-type transition state compared to the larger pores of HBEA. This leads to a higher reactivity with HMFI than with HBEA. However, this does not account for the observed differences in turnover frequency between HBEA and homogeneous HCl solution at high ionic strength. This discrepancy suggests that spatial constraints within HBEA micropores affect the reaction’s standard free energy pathway, which is discussed below.

### Elucidating the Intrinsic Catalytic Behavior of HBEA with Spatial Constraint

In the present section, we explore the reduction in catalytic activity observed beyond the optimal ionic strength. In contrast to homogeneous solutions that can expand their volume in the case of higher concentrations, cyclohexanol, and hydronium ions occupy a fixed limited space within the HBEA micropores. The hydrated hydronium ions and the negative charge located at aluminum tetrahedra are organized in fluxional polar (the hydronium ions themselves) and solvent-free domains, occupied by 1–2 cyclohexanol molecules. To estimate this space between hydrated hydronium ions, the remaining pore volume of HBEA was investigated by coadsorption of cyclohexanol and water, as shown in Figure S3.

**Figure 4:**
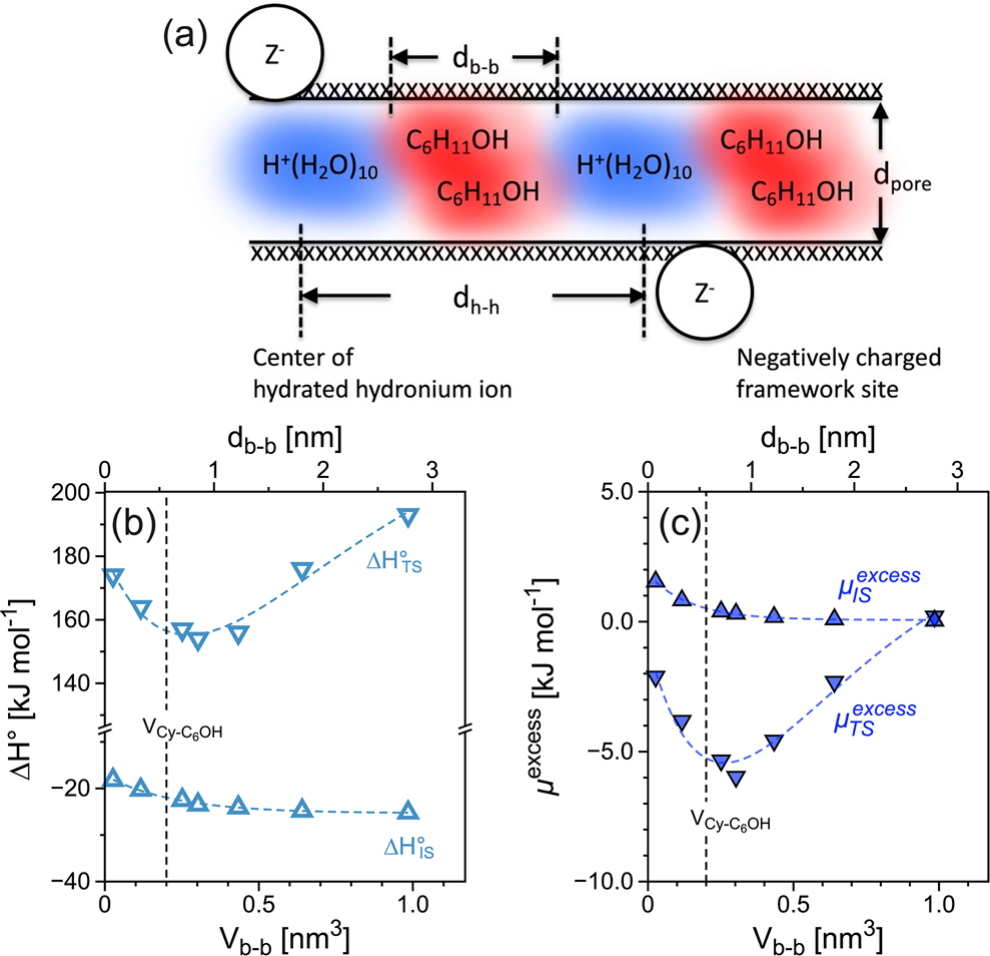
Impact of boundary distance of hydronium ions in the HBEA micropore on GS and TS energies: (a) local structure of the HBEA micropore with hydronium ions and cyclohexanol. (b) Enthalpy and (c) excess chemical potential of the ground and transition states as a function of the boundary distance (*d*_b–b_) between two neighboring hydronium ions, where *V*_b–b_ is calculated by a cylinder model with *d*_b–b_ and the 0.67 nm of pore diameter of the HBEA micropore. The dashed line represents the van der Waal (vdW) volume of one cyclohexanol molecule in the HBEA micropore.

Figure 4a shows a schematic representation of this local organization in the pores of HBEA. Hydrated hydronium ions form and ion pair with the negatively charged framework site. The average distance (*d*_h–h_) of such neighboring hydrated hydronium ions constitutes the space in which cyclohexanol can absorb with a volume of *V*_b–b_ and *d*_b–b_. By considering the composition of H_3_O_hydr_.^+^ in HBEA, i.e., H^+^(H_2_O)_10_, and taking a cylinder model, we determined that the length of H_3_O_hydr_.^+^ is 0.9 nm. The *d*_h–h_ and *d*_b–b_ values are different by the length of H_3_O_hydr_.^+^ in BEA, i.e., *d*_b–b_ = *d*_h–h_ – *L*_`_. The distances of *d*_h–h_ and *d*_b–b_ are derived from the BAS concentration (Figure S4a), which corresponds to the quantity of hydronium ions in the micropores, i.e., ionic strength (Figure S4b). The increase of BAS concentration thus leads to a decrease in the distance between hydronium ions, i.e., a decrease in *d*_h–h_ and *d*_b–b_. Considering the size of hydrated hydronium ions in HBEA, i.e., H^+^(H_2_O)_10_, *d*_h–h_ and *d*_b–b_ decrease from 3.8 to 1.1 nm and from 2.8 to 0.1 nm, respectively, with increasing BAS concentration from 0.02 to 0.82 mmol g^–1^. The respective *d*_h–h_ and *d*_b–b_ values in HBEA and HMFI are comparable at the same ionic strength (Figure S4b,c). The maximum rate was observed at 0.71 and 0.57 nm of boundary distance for HBEA and HMFI, respectively, where the ionic strength corresponds to respective 1.0 and 1.5 mol L^–1^ (Figure S5). This indicates the additional steric enhancement along with the optimum size of the transition state by neighboring hydronium ion clusters in HMFI compared to HBEA.

In our previous work on HMFI, we have shown that the space and distance between the boundaries of the hydrated hydronium ions are critical parameters that determine spatial constraints for sorbed cyclohexanol.^19^ The proximity of cyclohexanol to the polar domains of hydronium ions influences the enthalpy and excess chemical potential of the initial and transition states. Following the same concept, we investigated the variation of enthalpy and excess chemical potential in the initial and transition states as a function of the boundary distance (Figure 4b,c). Cyclohexanol has a stable enthalpy of the initial state (Δ*H*_IS_^°^ ≈ −23 kJ mol^–1^) with *d*_b–b_ larger than 0.7 nm, whereas it significantly increases to −18 kJ mol^–1^ below that threshold. This corresponds to the 4-fold increase of the excess chemical potential of the ground state (μ_IS_^excess^) from 0.4 to 1.6 kJ mol^–1^. In the transition state, both enthalpy (Δ*H*_TS_^°^) and excess chemical potential (μ_TS_^excess^) show a reverse-volcano trend, where the minimum values are observed at *d*_b–b_ of 0.7 nm, which corresponds to *V*_b–b_ of 0.25 nm^3^. In a simple geometric model of a cylinder of 0.67 nm micropore diameter, the van der Waal (vdW) volume of one cyclohexanol molecule occupies 0.20–0.21 nm^3^, i.e., 0.57–0.60 nm length, at 150–180 °C. The lowest Δ*H*_TS_^°^ and μ_TS_^excess^ are reached when *V*_b–b_ or *d*_b–b_ is close to the volume or length of a cyclohexanol molecule.

On the other hand, the increase of Δ*H*_TS_^°^ and μ_TS_^excess^ at lower *V*_b–b_ or *d*_b–b_ is ascribed to additional spatial constraints induced on the hydrated hydronium ions. Figure S5 shows the TOFs of HMFI and HBEA as a function of *d*_b–b_. The TOF decreased when *d*_b–b_ was shorter than the diameter of the micropores. Moreover, the dehydration of substituted cyclohexanols, such as 4-methylcyclohexanol and *cis*-2-methylcyclohexanol, via their respective E1 (stepwise) and E2 (concerted) pathways, exhibits volcano-like dependencies on ionic strength in HMFI. Notably, a consistent decrease in reaction rate is observed below a critical boundary distance of 0.4 nm.^20^

The spatial constraint at higher ionic strength, therefore, induces a rearrangement of cation and anion pairs for H^+^(H_2_O)_*n*_-zeolite (HMFI or HBEA), leading to partly compensating the reduction of the free-energy barrier by the excess chemical potential in initial and transition states, regardless of zeolite geometry and dehydration mechanism. In comparison, the reaction catalyzed by homogeneous HCl has an identical impact of the ionic strength but does not experience the spatial constraints. Higher ionic strength will then simply lead to a minor expansion of the liquid volume without penalty of steric rearrangements or separations of charge (Figure S6a).

### Correlation of the Hydronium Ion-Catalyzed Dehydration of Cyclohexanol with the Standard Chemical Potentials in Initial and Transition States

The volcano-like correlation between the reaction rates and the hydronium-ion-derived ionic strength in the micropore was observed for both HMFI and HBEA (Figure S6a). Thus, the compromise between activity enhancement and rearrangement of cation and anion pairs in the pore by spatial limitation appears to be a general feature of zeolites. The profiles of activation enthalpy and entropy as a function of ionic strength on HBEA are shifted toward more positive (Δ*H*_TS_^°^) and lower ionic strength compared to HMFI (Figure S6b,c). This is ascribed to the larger pore diameter of HBEA (0.67 nm) than HMFI (0.55 nm), which leads to lower van der Waals stabilization of the transition state.

**Figure 5:**
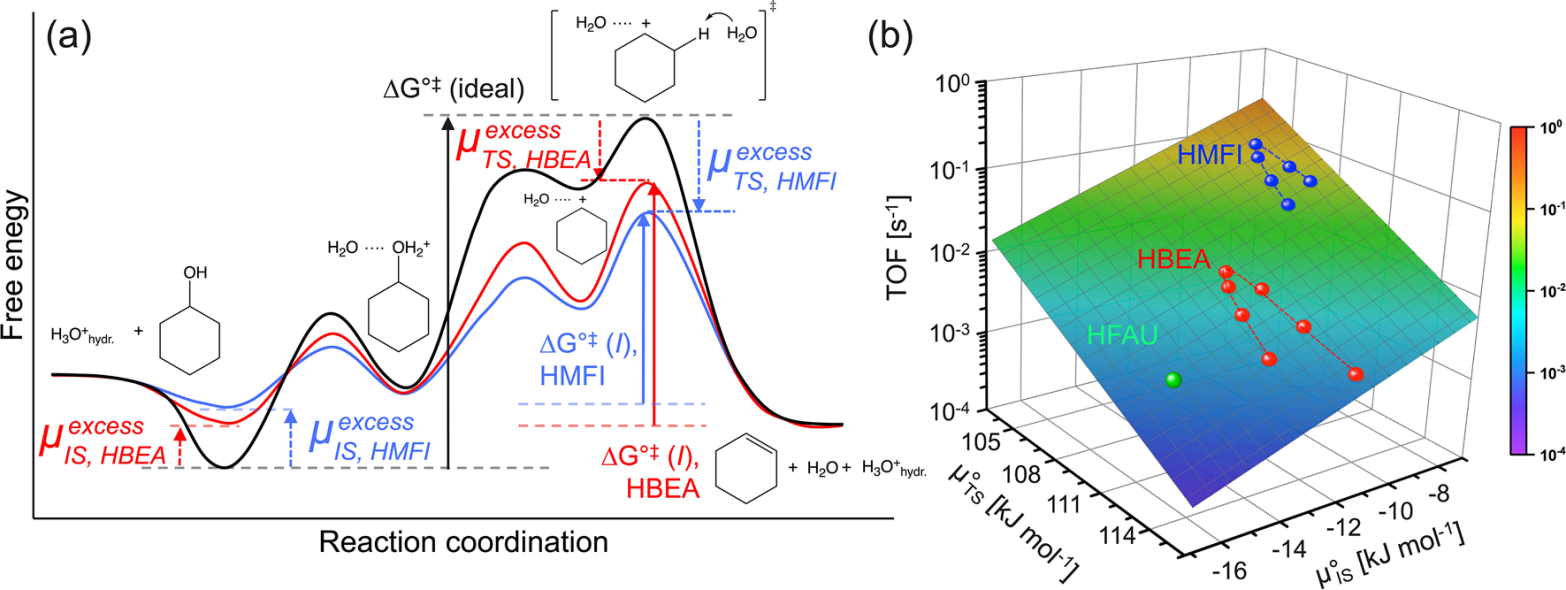
Free-energy barrier of hydronium ion-catalyzed cyclohexanol dehydration: (a) free energies of the elementary steps for dehydration of cyclohexanol on hydronium ions in HMFI and HBEA. (b) Reaction rate of cyclohexanol dehydration in water on HMFI,^19^ HBEA, and HFAU^9^ as a function of the correlation between chemical potentials in the initial and transition states at 150 °C. The TOF of H-MFI in (b) is reproduced with permission from ref (19). Copyright 2021, AAAS.

The enhancement of catalytic activity is influenced by how well the reacting molecule, including its initial and transition states, fits within the microporous environment. The ionic environment contributes to the destabilization of adsorbed cyclohexanol in its initial state while stabilizing the carbenium ion in the transition state, as illustrated in Figure 5a. The respective chemical potentials in the initial and transition states at 150 °C were assessed to be −8 and 105 kJ mol^–1^ for HMFI and −12 and 109 kJ mol^–1^ for HBEA at each optimum ionic strength, whereas it was −16 and 113 kJ mol^–1^ for HFAU, respectively.^9^ The more restricted pore space of HMFI results in a larger entropy loss, leading to less effective adsorption of cyclohexanol compared with HBEA and HFAU, reflected by a higher chemical potential in the initial state. By considering the comparable excess chemical potential in the initial state between HMFI and HBEA, it is indicated that the transition state of the cyclohexanol carbenium ion for dehydration is more effectively stabilized in HMFI’s smaller pores. Therefore, evaluating the chemical and excess chemical potentials in both the initial and transition states allows us to anticipate a higher reactivity of HMFI for the hydronium ion-catalyzed dehydration of cyclohexanol. This is in line with the TOFs as a function of the energy barrier in the ground and transition states (Figure 5b), resulting in orders of magnitude increase of TOF for cyclohexanol dehydration over HMFI compared to HBEA and HFAU at 150 °C.

## Conclusions

Hydronium ions, formed in the presence of water along with the negatively charged framework, create an ionic environment within the micropores of zeolites. The resulting ionic strength significantly affects the rate of dehydration of cyclohexanol within these micropores. Specifically, the transition states are stabilized, while the initial states are destabilized, resulting in a decrease in the free-energy barrier for hydronium ion-catalyzed dehydration. However, high concentrations of hydronium ions necessitate the rearrangement of the hydronium ions and lead to increased charge separation of the cation and anion pairs, i.e., H^+^(H_2_O)_*n*_ and the negatively charged AlO_4_ lattice tetrahedron. This rearrangement increases the standard free energy and excess chemical potential of the reacting alcohol in the transition state. The volcano-type correlation observed between turnover frequencies (TOFs) and ionic strength, initially found for HMFI zeolites and confirmed here with HBEA zeolites, arises from the compensation of reactivity enhancement induced by ionic strength with the rearrangement required at higher ionic strength levels. The van der Waals stabilization of the transition state within the narrower pores of HMFI provides better standard free energy stabilization of the carbenium-ion-type transition state compared to the wider pores of HBEA, making HMFI more active than HBEA. These findings illustrate that modifying the molecular environment within the micropores can lead to significant rate enhancements under mild conditions. The presented generalized model allows for the estimation and prediction of reaction rates via defining the standard chemical potential in both the initial and transition states.

## Supporting Information Available

Experimental methods, calculation of hydronium ions in HBEA, free energy barrier and excess chemical potential estimation at initial and transition states for hydronium ion-catalyzed cyclohexanol dehydration, TOF in cyclohexanol dehydration in HBEA via partition function analysis, and reaction rate of cyclohexanol dehydration and adsorption isotherm of aqueous cyclohexanol over HBEA (PDF)
