# Supplementary material for: Confined Ionic Environments Tailoring the Reactivity of Molecules in the Micropores of BEA-Type Zeolite
Source: J Am Chem Soc. 2024 Jun 18;146(26):17847–53. doi: 10.1021/jacs.4c03405 (PMC11228971; doi:10.1021/jacs.4c03405)
Supplement: Supplementary file 1 — ja4c03405_si_001.pdf [file ja4c03405_si_001.pdf]

Supporting information

Confined Ionic environments tailoring the reactivity of molecules in the micropores of BEA-type zeolite

Sungmin Kim[a], Feng Chen[a], Donald M. Camaioni[a], Miroslaw A.Derewinski[a], Oliver Y. Gutiérrez[a], Yue Liu[b], Johannes A. Lercher[a,b]

[a]Institute for Integrated Catalysis and Physical Science Division, Pacific Northwest National Laboratory, Richland, Washington 99354, United States
[b]Department of Chemistry and Catalysis Research Institute, TU München, Lichtenbergstrasse 4, 85748 Garching, Germany

S. 1. Experimental method

Chemicals

Octadecyltrichlorosilane (Sigma-Aldrich, > 90%), Cyclohexanol (Sigma-Aldrich, 99%), cyclohexene (Sigma-Aldrich, 99%, GC-grade), 1,3-dimethoxy-benzene (Sigma-Aldrich, 99%), dichloromethane (Sigma-Aldrich, HPLC grade), and sodium sulfate (Acros Organics, 99%, anhydrous) were used as received without further purification.

Preparation of low-defect HBEA

The low-defect HBEA zeolite with different Si/Al ratios (from 15 to 400) was synthesized in the presence of fluoride. The detail procedure have been reported elsewhere.[1, 2] 6, 13 In brief, BEA zeolite was prepared by hydrothermal synthesis at 140 °C, with a gel composition of SiO_2 : xAl_2O_3 : (0.54 + 2x) TEAOH : (0.54 + 2x) HF : (7 + 2x) H_2O. Subsequently, the collected material was washed and filtrated with deionized water until pH neutral. To decompose the residues, e.g., organic cation (TEA+) and remove fluoride anions, the dried material was calcined at 500 °C for 2 h in synthetic air.

Characterization

N_2-physisorption The specific surface area and pore size distribution of prepared HBEA were measured by N_2-physisorption at -196 °C using Micrometrics ASAP 2020. Prior to N_2 adsorption, each sample was degassed at 300 °C for 5 h under vacuum (10^{-3} mbar). The BET, BJH and t-plot methods were employed to determine the specific surface area, mesopore volume and micropore volume, respectively.

Infrared (IR) spectroscopy A self-supporting disc of HBEA with a density of approximately 10 mg/cm^2 was loaded in the *in situ* IR cell. The sample disc was evacuated (10^{-6} mbar) and heated to 450 °C with a heating rate of 10 °C/min and kept for 1 h. Then the sample was cooled to 150 °C for collection of IR spectra. All IR spectra were recorded on a ThermoScientific Nicolete FTIR spectrometer using a MCT detector with a resolution of 4 cm^{-1} and accumulated for 512 scans. The acid site concentration was determined by IR spectra of pyridine adsorbed on the HBEA sample. After equilibration with pyridine vapors at 0.1 mbar (0.5–1.0 h) at 150 °C, all physisorbed pyridine was outgassed under vacuum (<10^{-6} mbar). The integral area of the Brønsted (1565–1515 cm-1) and Lewis peaks (1470–1430 cm-1) with the molar extinction coefficients (0.73 cm/μmol

and 0.96 cm/μmol for BAS and LAS, respectively) and disc weight were employed for quantification of acid site concentration.

Adsorption measurement for aqueous-phase cyclohexanol on HBEA

The aqueous phase adsorption of cyclohexanol on HBEA was carried out by immersing 30–40 mg HBEA in an aqueous solution of cyclohexanol at a certain concentration and agitation (≈ 700 rpm) for at least 24 h. The zeolite was filtered from the liquid and the residual concentration of cyclohexanol in the filtrate was measured with Agilent 7890A GC equipping a flame ionization detector (FID) and HP-5 column (25 m of length and 0.25 μm i.d.) after extraction with dichloromethane. The quantification was done using 1,3-dimethoxy-benzene as an internal standard. The uptake was determined by the change in bulk concentration, volume of the solution and mass of the solid sample as following:

$$Q = Q_{sat}\frac{K^{\circ}_{ads}[Cy - C_6OH]_{aq}}{1 + K^{\circ}_{ads}[Cy - C_6OH]_{aq}} \qquad \text{(S1)}$$

where Q is the cyclohexanol uptake; Q_{sat} is saturated uptake of cyclohexanol; $[Cy\text{-}C_6OH]_{\text{aq}}$ is the equilibrium concentration of aqueous cyclohexanol; K°_{ads} is the adsorption constant. The coverage of cyclohexanol (θ) can be expressed by the quotient of Q/Q_{sat} ($\theta = Q/Q_{\text{sat}}$).

Kinetic measurements:

Kinetic measurements were performed at 150-180 °C using a 300 ml Hastelloy PARR reactor. In typical reaction, aqueous cyclohexanol solution (0.3 M, 100 ml) and 200 mg HBEA zeolite (Si/Al = 15–400). The reactor was then pressurized with 40 bar H_2 at room temperature and heated to reaction temperature (150–180 °C) under vigorous stirring at 700 rpm. After the reaction, the reactor was immediately cooled below 5 °C with an ice/water mixture. The organic phase was extracted with 150 ml dichloromethane (30 ml per extraction, 5 times) and dried over sodium sulfate. The reactant and products were quantified with external standard of 1,3-dimethoxybenzene using an Agilent 7890A GC equipped with a flame ionization detector (FID) and HP-5MS column (25 x 0.25 μm i.d).

HCl-catalyzed dehydration of cyclohexanol was performed in a 100 ml Hastelloy Parr reactor. In a typical experiment, 33 mL aqueous solution of 0.001 M HCl, 0.33 M cyclohexanol and different concentrations of LiCl were loaded in the reactor. The reactor was sealed and purged

three times with 40 bar N_2, and afterwards pressurized to a N_2-pressure of 40 bar at ambient temperature. The reactor was then heated to desired reaction temperatures, e.g. 180 °C. As soon as the temperature was reached, agitation (700 rpm) was started. At the end of a reaction, the reactor was immediately cooled with an ice/water mixture to 5 °C and lower. Reactants and products were extracted three times with 20 ml of ethyl acetate each time, and 2-cyclohexen-1-one was used as an internal standard. The organic phase, was then separated and dried over sodium sulfate, and analyzed on a Shimadzu 2010 GC equipped with an HP-5MS 25m×0.25 mm (i.d.) column and a flame ionization detector (FID).

Therefore, to quantify the concentration of hydronium ions, 33 mL of the recovered solution was added to the 100 mL Hastelloy Parr reactor and cyclohexanol (0.33 M) was added. Multiple reactions with HCl as a catalyst were conducted (conversion < 10%) to allow calculating rates of HCl-catalyzed cyclohexene formation.

S. 2. Supplementary notes

S. 2. 1. Determination of the number of water molecules constituting a hydronium ion in HBEA

The number of water molecules (n_{H_2O}) constituting a hydronium ion in HBEA was determined by the approach described in a previous work.[3] In brief, the volume occupied by hydronium was calculated from the difference between the micropore volume of HBEA (V_{micro}) and the volume of a substrate, e.g. cyclohexanol, determined by the saturated uptake (Q_{sat}) on HBEA in the aqueous phase:

$$V_{sub} + V_{hydr} = V_{micr} \qquad \text{(S2)}$$

By considering the saturated adsorption, the adsorbed substrate and hydronium ions completely occupied the HBEA micropores. Hence, the volume of hydronium ions would be proportional to its concentration in micropores (Eq. S3).

$$V_{hydr} = n_{H_2O} \cdot V_{m,H_2O} \cdot c_{BAS} \qquad \text{(S3)}$$

where, V_{m,H_2O} is the molar volume of water, which is assumed that water in the hydronium ion has the same packing density as that of free water; c_{BAS} is the BAS concentration of HBEA. Therefore, the calculated V_{hydr} showed a linear correlation with c_{BAS} on HBEA (Figure S4b). Then n_{H_2O} is obtained from the quotient of the slope to V_{m,H_2O} (Eq. S3).

S. 2. 2. Calculation of free energy barrier and excess chemical potential of initial and transition state for hydronium ion-catalyzed cyclohexanol dehydration in HBEA.

The activity coefficient of adsorbed cyclohexanol increases exponentially with ionic strength, which is quantifiable by a salting-out model:

$$\log_{10}\left(\gamma_{\mathrm{Cy,ad}}\right) = K_{\mathrm{s}} I_{\mathrm{HBEA}} \qquad \text{(S4)}$$

in which K_s is the Setschenow constant, which is determined from the correlation between BAS concentration and adsorption constant of cyclohexanol in water (Figure S4c). The standard state chemical potential of adsorbed cyclohexanol is a function of ionic strength (I_{HBEA}) in HBEA, $\mu^{\mathrm{o}}_{\mathrm{Cy,ad}}(I_{\mathrm{HBEA}})$, expressed as

$$\mu^{0}_{\mathrm{Cy,ad}}(I_{\mathrm{HBEA}}) = \mu^{0}_{\mathrm{Cy,ad}}(0) + RT\ln\gamma_{\mathrm{Cy,ad}} = \mu^{0}_{\mathrm{Cy,ad}}(0) + 2.303 \times RTK_{\mathrm{s}} I_{\mathrm{HBEA}} \qquad \text{(S5)}$$

The $\mu^{\mathrm{o}}_{\mathrm{Cy,ad}}(0)$ is the chemical potential of cyclohexanol adsorbed in HBEA zeolite free of ions (I = 0), e.g. purely siliceous BEA. For the adsorption of cyclohexanol from the aqueous phase in HBEA with an ionic strength of I_{HBEA}, the adsorption constant $K^{\mathrm{o}}_{\mathrm{ad}}$ is expressed as

$$K^{\mathrm{o}}_{\mathrm{ad}} = \exp\left[-\frac{\mu^{0}_{\mathrm{Cy,ad}}(I_{\mathrm{HBEA}}) - \mu^{0}_{\mathrm{Cy,aq}}}{RT}\right] = \exp\left[-\frac{\mu^{0}_{\mathrm{Cy,ad}}(0) - \mu^{0}_{\mathrm{Cy,aq}}}{RT}\right]\cdot 10^{-K_{\mathrm{s}} I_{\mathrm{HBEA}}} \qquad \text{(S6)}$$

This equation predicts that $K^{\mathrm{o}}_{\mathrm{ad}}$ increases exponentially with the I_{HMFI}.

Based on the Gibbs-Helmholtz equation, the adsorption heat ΔQ_{ad} and enthalpy $\Delta H^{\mathrm{o}}_{\mathrm{ad}}$ are given by equation (S7).

$$\frac{\Delta Q_{\mathrm{ad}}}{T^2} = -\frac{\Delta H^{\mathrm{o}}_{\mathrm{ad}}}{T^2} = \left(\frac{\partial\left(\Delta G^{\mathrm{o}}_{\mathrm{ad}}/T\right)}{\partial T}\right)_p = -\left(\frac{\partial\left(R\ln K^{\mathrm{o}}_{\mathrm{ad}}\right)}{\partial T}\right)_p \qquad \text{(S7)}$$

Under constant temperature and pressure, using the expression of $K^{\mathrm{o}}_{\mathrm{ad}}$ in Equation S6 allows Equation S6 to be reformulated as following:

$$\Delta Q_{\mathrm{ad}} = -\frac{\mathrm{d}\left(\left(\mu^{0}_{\mathrm{Cy,ad}}(0) - \mu^{0}_{\mathrm{Cy,aq}}\right)/T\right)}{\mathrm{d}\left(1/T\right)} - 2.303\cdot RI_{\mathrm{HBEA}}\cdot\frac{\mathrm{d}K_{\mathrm{s}}}{\mathrm{d}\left(1/T\right)} \qquad \text{(S8)}$$

$$\frac{\mathrm{d}K_{\mathrm{s}}}{\mathrm{d}\left(1/T\right)} = -\frac{1}{2.303\cdot R}\cdot\frac{\mathrm{d}\Delta Q_{\mathrm{ad}}}{dI_{\mathrm{HBEA}}} \qquad \text{(S9)}$$

Hence, equation S8 gives the variation of K_s with T.

Figure S4c shows the measured K_{ad}^{o} and ΔQ_{ad} of cyclohexanol on HBEA with different ionic strength at 298 K. By regression of the two plots using equations S6 and S9, K_s at 298 K is determined to be 0.12 ± 0.2 and $dK_s/d(1/T)$ is 108 ± 7 K. Assuming that $dK_s/d(1/T)$ is invariant with temperature, the K_s at 423 K is calculated to be 0.02 ± 0.01.

$$K_s(423\ \mathrm{K}) = K_s(298\ \mathrm{K}) + \frac{\mathrm{d}K_s}{\mathrm{d}(1/T)} \cdot \left(\frac{1}{423\ \mathrm{K}} - \frac{1}{298\ \mathrm{K}} \right) = 0.02 \pm 0.01 \qquad \text{(S10)}$$

Thus the excess chemical potential of sorbed cyclohexanol in HBEA as reacting initial state (μ_{IS}^{excess}) is calculated by

$$\mu_{GS}^{excess} = 2.303 \times RTK_s I \qquad \text{(S11)}$$

The excess chemical potential of transition state (μ_{TS}^{excess}) is calculated by

$$\mu_{TS}^{excess} = \Delta G^{\circ\ddagger}(I) - \Delta G^{\circ\ddagger}_{(ideal)} + \mu_{GS}^{excess} \qquad \text{(S12)}$$

The $\Delta G^{o\ddagger}{}_{(ideal)}$ and $\Delta G^{o\ddagger}(I)$ are the free energy barrier under ideal condition and in the presence of an ionic strength, respectively. They are obtained from the measured reaction TOF using transition state formula.

$$\mathrm{TOF}_{(ideal)} = \frac{k_B T}{h} \exp\left(-\frac{\Delta G^{\circ\ddagger}_{(ideal)}}{RT} \right) \qquad \text{(S13)}$$

$$\mathrm{TOF}(I) = \frac{k_B T}{h} \exp\left(-\frac{\Delta G^{\circ\ddagger}(I)}{RT} \right) \qquad \text{(S14)}$$

The $\mathrm{TOF}_{(ideal)}$ under the ideal condition was obtained as 0.001 s^{-1} by extrapolating the TOF to zero ionic strength, because it is experimentally impossible to measure the reaction rate of an HBEA with zero ionic strength, i.e., without BAS.

Taking the relation of $\Delta G^{o\ddagger}{}_{(ideal)}$ and $\Delta G^{o\ddagger}(I)$ in Equation S12 into Equation S14, gives

$$\mathrm{TOF}(I) = \frac{k_{\boldsymbol{B}} T}{h} \exp\left(-\frac{\Delta G^{\circ\ddagger}(\mathrm{I})}{RT} \right) = \frac{k_{\boldsymbol{B}} T}{h} \exp\left(-\frac{\Delta G^{\circ\ddagger}_{(ideal)} + \mu_{TS}^{excess} - \mu_{IS}^{excess}}{\mathrm{RT}} \right)$$

$$= \frac{k_{\boldsymbol{B}} T}{h} \exp\left(-\frac{\Delta G^{\circ\ddagger}_{(ideal)}}{RT} \right) \left(-\frac{\mu_{TS}^{excess}}{RT} \right) \left(\frac{\mu_{IS}^{excess}}{RT} \right) = \mathrm{TOF}_{(ideal)} \cdot \frac{\gamma_{IS}(I)}{\gamma_{TS}(I)} \qquad \text{(S15)}$$

S. 2. 3. *Calculation of TOF in cyclohexanol dehydration in HBEA via partition function analysis.*

The cyclohexanol (A for short) adsorbs in zeolite pore channels, reacting through a transition state (‡) to the product. Assuming there are N adsorption sites in the pore channel and no interaction between adsorbed species. When N_A amount of cyclohexanol adsorbed and $N^‡$ amount of transition state exist for the reaction ($N_A >> N^‡$), the canonical partition function $Q(N, N_A, N^‡)$ at constant temperature is expressed as:

$$Q\left(N,N_{\mathrm{A}},N^{‡}\right)=\frac{N!}{N_{\mathrm{A}}!N^{‡}!(N-N_{\mathrm{A}}-N^{‡})!}\cdot q_{\mathrm{A,p}}^{N_{\mathrm{A}}}\cdot q_{‡,\mathrm{p}}^{N^{‡}} \quad \text{(S16)}$$

where $q_{A,p}$ and $q_{‡,p}$ are the molecular partition function of A and reacting transition state in the pore, respectively. Then the chemical potential of A ($\mu_{A,p}$) and transition state ($\mu_{‡,p}$) are expressed:

$$\mu_{\mathrm{A,p}}=-k_{\mathrm{B}}T\frac{\partial\ln Q\left(N,N_{\mathrm{A}},N^{‡}\right)}{\partial N_{\mathrm{A}}}=-k_{\mathrm{B}}T\ln q_{\mathrm{A,p}}-k_{\mathrm{B}}T\frac{\partial\left[\ln N!-\ln N_{\mathrm{A}}!-\ln N^{‡}!-\ln(N-N_{\mathrm{A}}-N^{‡})!\right]}{\partial N_{\mathrm{A}}} \quad \text{(S17)}$$

$$\mu_{‡,\mathrm{p}}=-k_{\mathrm{B}}T\frac{\partial\ln Q\left(N,N_{\mathrm{A}},N^{‡}\right)}{\partial N^{‡}}=-k_{\mathrm{B}}T\ln q_{‡,\mathrm{p}}-k_{\mathrm{B}}T\frac{\partial\left[\ln N!-\ln N_{\mathrm{A}}!-\ln N^{‡}!-\ln(N-N_{\mathrm{A}}-N^{‡})!\right]}{\partial N^{‡}} \quad \text{(S18)}$$

Applying Stirling approximation, eq. S17 and S18 is transformed into eq. S19 and S20:

$$\begin{aligned}\mu_{\mathrm{A,p}}&=-k_{\mathrm{B}}T\ln q_{\mathrm{A,p}}-k_{\mathrm{B}}T\frac{\partial}{\partial N_{\mathrm{A}}}\left[N\ln N-N_{\mathrm{A}}\ln N_{\mathrm{A}}-N^{‡}\ln N^{‡}-(N-N_{\mathrm{A}}-N^{‡})\ln(N-N_{\mathrm{A}}-N^{‡})\right]\\&=-k_{\mathrm{B}}T\ln q_{\mathrm{A,p}}-k_{\mathrm{B}}T\left[-\ln N_{\mathrm{A}}-1+\ln(N-N_{\mathrm{A}}-N^{‡})+1\right] \quad \text{(S19)}\\&=-k_{\mathrm{B}}T\ln q_{\mathrm{A,p}}+k_{\mathrm{B}}T\ln\frac{N_{\mathrm{A}}}{N-N_{\mathrm{A}}-N^{‡}}\end{aligned}$$

$$\begin{aligned}\mu_{‡,\mathrm{p}}&=-k_{\mathrm{B}}T\ln q_{‡,\mathrm{p}}-k_{\mathrm{B}}T\frac{\partial}{\partial N^{‡}}\left[N\ln N-N_{\mathrm{A}}\ln N_{\mathrm{A}}-N^{‡}\ln N^{‡}-(N-N_{\mathrm{A}}-N^{‡})\ln(N-N_{\mathrm{A}}-N^{‡})\right]\\&=-k_{\mathrm{B}}T\ln q_{‡,\mathrm{p}}-k_{\mathrm{B}}T\left[-\ln N^{‡}-1+\ln(N-N_{\mathrm{A}}-N^{‡})+1\right] \quad \text{(S20)}\\&=-k_{\mathrm{B}}T\ln q_{‡,\mathrm{p}}+k_{\mathrm{B}}T\ln\frac{N^{‡}}{N-N_{\mathrm{A}}-N^{‡}}\end{aligned}$$

Number of transition states N‡ are very small ($N_A >> N^‡$). So the $\mu_{A,p}$ and $\mu_{‡,p}$ are further expressed:

$$\mu_{\mathrm{A,p}}=-k_{\mathrm{B}}T\ln q_{\mathrm{A,p}}+k_{\mathrm{B}}T\ln\frac{N_{\mathrm{A}}}{N-N_{\mathrm{A}}} \quad \text{(S21)}$$

$$\mu_{‡,\mathrm{p}}=-k_{\mathrm{B}}T\ln q_{‡,\mathrm{p}}+k_{\mathrm{B}}T\ln\frac{N^{‡}}{N-N_{\mathrm{A}}} \quad \text{(S22)}$$

Coverage (θ) is often used to describe an adsorption process. In this particular case the coverage of A is defined as $\theta_{\mathrm{A}} = N_{\mathrm{A}} / N$; and that of transition state is defined as $\theta^{\ddagger} = N^{\ddagger} / N$. The eq. S21 and S22 can be then expressed:

$$\mu_{\mathrm{A,p}} = -k_{\mathrm{B}}T\ln q_{\mathrm{A,p}} + k_{\mathrm{B}}T\ln\frac{\theta_{\mathrm{A}}}{1-\theta_{\mathrm{A}}} \tag{S23}$$

$$\mu_{\ddagger,\mathrm{p}} = -k_{\mathrm{B}}T\ln q_{\ddagger,\mathrm{p}} + k_{\mathrm{B}}T\ln\frac{\theta^{\ddagger}}{1-\theta_{\mathrm{A}}} \tag{S24}$$

Eq. S23 and S24 are the chemical potential under ideal condition. When accounting the interactions under non-ideal conditions, it is corrected by an activity coefficient term, $\gamma_{\mathrm{A,p}}$ or $\gamma_{\ddagger,\mathrm{p}}$

$$\mu_{\mathrm{A,p}} = -k_{\mathrm{B}}T\ln q_{\mathrm{A,p}} + k_{\mathrm{B}}T\ln\frac{\theta_{\mathrm{A}}}{1-\theta_{\mathrm{A}}} + k_{\mathrm{B}}T\ln\gamma_{\mathrm{A,p}} \tag{S25}$$

$$\mu_{\ddagger,\mathrm{p}} = -k_{\mathrm{B}}T\ln q_{\ddagger,\mathrm{p}} + k_{\mathrm{B}}T\ln\frac{\theta^{\ddagger}}{1-\theta_{\mathrm{A}}} + k_{\mathrm{B}}T\ln\gamma_{\ddagger,\mathrm{p}} \tag{S26}$$

Consider a catalyst having local ionic strength (I) coexist with an ideal catalyst (I = 0). Under equilibrium, A and transition state in both catalysts have the same chemical potential, respectively:

$$-k_{\mathrm{B}}T\ln q_{\mathrm{A,p}} + k_{\mathrm{B}}T\ln\frac{\theta_{\mathrm{A}}(I)}{1-\theta_{\mathrm{A}}(I)} + k_{\mathrm{B}}T\ln\gamma_{\mathrm{A,p}}(I) = -k_{\mathrm{B}}T\ln q_{\mathrm{A,p}} + k_{\mathrm{B}}T\ln\frac{\theta_{\mathrm{A}}(0)}{1-\theta_{\mathrm{A}}(0)} \tag{S27}$$

$$-k_{\mathrm{B}}T\ln q_{\ddagger,\mathrm{p}} + k_{\mathrm{B}}T\ln\frac{\theta^{\ddagger}(I)}{1-\theta_{\mathrm{A}}(I)} + k_{\mathrm{B}}T\ln\gamma_{\ddagger,\mathrm{p}}(I) = -k_{\mathrm{B}}T\ln q_{\ddagger,\mathrm{p}} + k_{\mathrm{B}}T\ln\frac{\theta^{\ddagger}(0)}{1-\theta_{\mathrm{A}}(0)} \tag{S28}$$

The I in parentheses represents the ionic strength in the zeolite, while the value of 0 refers to the ideal zeolite. The Eq. S27 and S28 is reformulated to

$$k_{\mathrm{B}}T\ln\frac{\theta_{\mathrm{A}}(I)}{\theta_{\mathrm{A}}(0)} + k_{\mathrm{B}}T\ln\gamma_{\mathrm{A,p}}(I) = k_{\mathrm{B}}T\ln\frac{1-\theta_{\mathrm{A}}(I)}{1-\theta_{\mathrm{A}}(0)} \tag{S29}$$

$$k_{\mathrm{B}}T\ln\frac{\theta^{\ddagger}(I)}{\theta^{\ddagger}(0)} + k_{\mathrm{B}}T\ln\gamma_{\ddagger,\mathrm{p}}(I) = k_{\mathrm{B}}T\ln\frac{1-\theta_{\mathrm{A}}(I)}{1-\theta_{\mathrm{A}}(0)} \tag{S30}$$

Noticing that the right part of Eq. S29 and S30 are the same, therefore their left parts equal.

$$k_{\mathrm{B}}T\ln\frac{\theta_{\mathrm{A}}(I)}{\theta_{\mathrm{A}}(0)} + k_{\mathrm{B}}T\ln\gamma_{\mathrm{A,p}}(I) = k_{\mathrm{B}}T\ln\frac{\theta^{\ddagger}(I)}{\theta^{\ddagger}(0)} + k_{\mathrm{B}}T\ln\gamma_{\ddagger,\mathrm{p}}(I) \tag{S31}$$

This gives the expression of transition state ratio

$$\frac{q^{\ddagger}(I)}{q^{\ddagger}(0)} = \frac{q_{\mathrm{A}}(I)}{q_{\mathrm{A}}(0)} \times \frac{g_{\mathrm{A,p}}(I)}{g_{\ddagger,\mathrm{p}}(I)} \tag{S32}$$

Under reaction order of zero, zeolite pore is saturated by A, i.e. $\theta_{\mathrm{A}}(I) = \theta_{\mathrm{A}}(0) = 1$. The ratio of $\theta^{\ddagger}(I)$ / $\theta^{\ddagger}(0)$ is the ratio of TOF(I) / TOF(0), because transition state has a constant frequency to convert to product. So Equation S32 transforms into

$$\frac{\mathrm{TOF}(I)}{\mathrm{TOF}(0)} = \frac{g_{\mathrm{A,p}}(I)}{g_{\ddagger,\mathrm{p}}(I)} \tag{S33}$$

$$\mathrm{TOF}(I) = \mathrm{TOF}(0) \times \frac{g_{\mathrm{A,p}}(I)}{g_{\ddagger,\mathrm{p}}(I)} \tag{S34}$$

S. 3. Supplementary figures

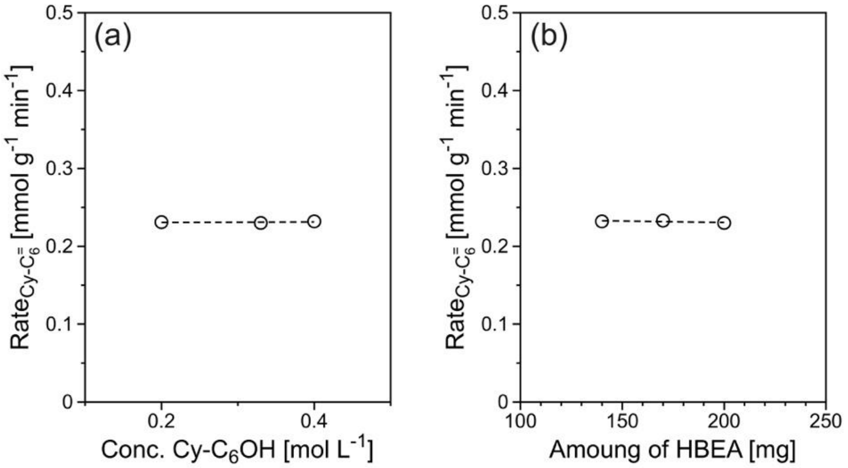

Figure S1. Cyclohexene formation rate on HBEA (Si/Al = 75) at 160 °C as a function of (a) the amount of HBEA and (b) the concentration of aqueous cyclohexanol.

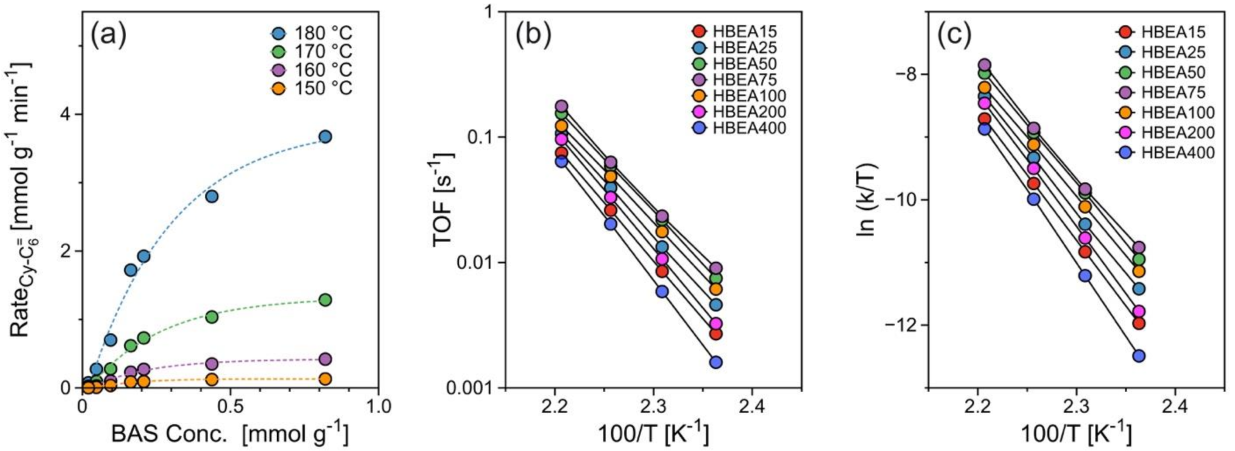

Figure S2. (a) Reaction rate of aqueous-phase dehydration of cyclohexanol as a function of BAS concentration of HBEA (Si/Al = 15–200) at 150–180 °C. (b) Arrhenius and (c) Eyring plots for aqueous-phase dehydration of cyclohexanol.

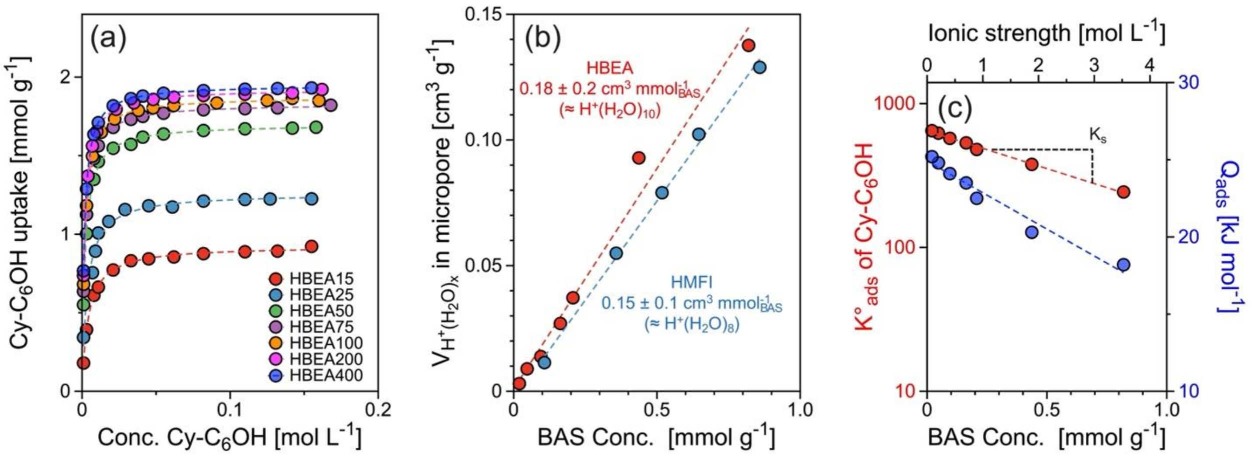

Figure S3. (a) Adsorption isotherm of aqueous-phase cyclohexanol on HBEA (Si/Al = 15–400) at room temperature. (b) The volume of hydrated hydronium ions in the micropore of HBEA and HMFI[3] as a function of BAS concentration. (c) The adsorption constant (K^{o}_{ads}) and adsorption heat (Q_{ads}) of aqueous cyclohexanol on HBEA at room temperature as a function of BAS concentration (or ionic strength)[4]. Figure S3 is reproduced with permission from Ref. 3 and 4. Copyrights of 2019 John Wiley and Sons, and 2023 American Chemical Society, respectively.

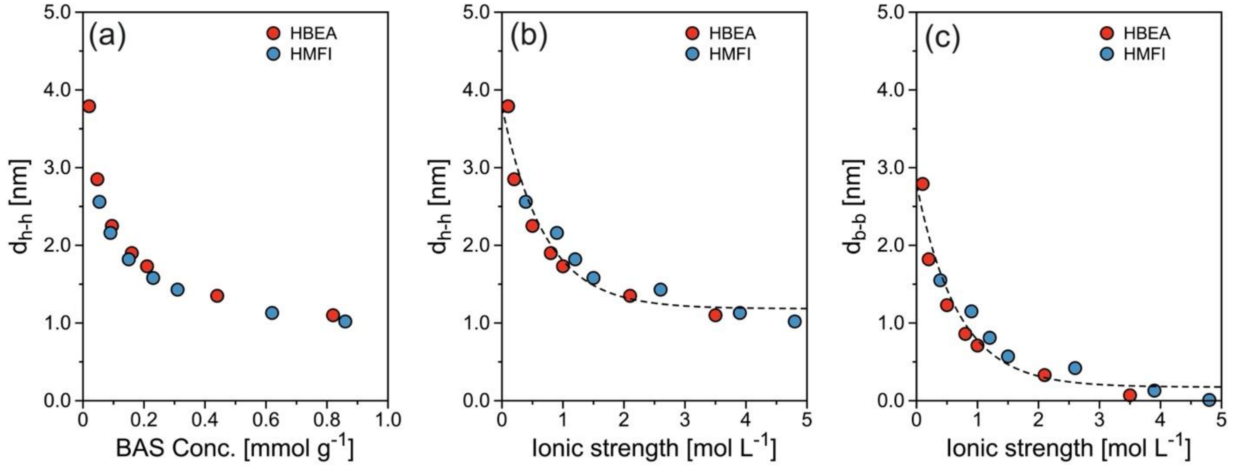

Figure S4. The average distance between two neighboring hydronium ion clusters ($d_{h\text{-}h}$) as a function of (a) Brønsted acid site concentration and (b) ionic strength in HBEA and HMFI. (c) boundaries ($d_{b\text{-}b}$) of hydronium ion clusters in HBEA and HMFI as a function of ionic strength.

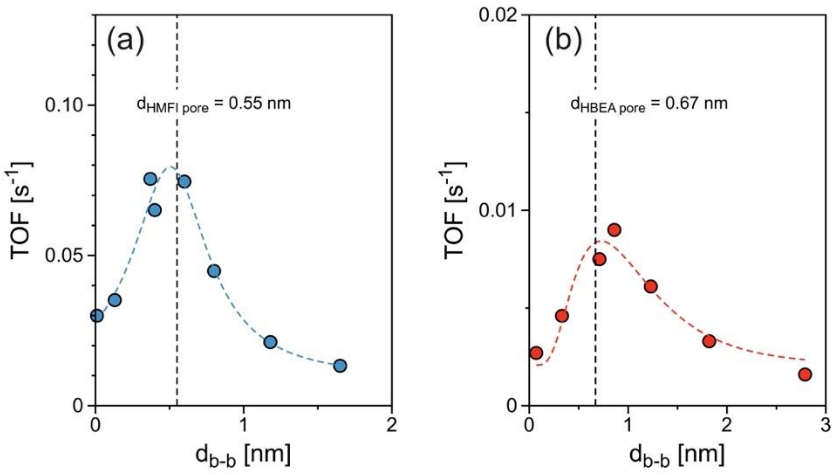

Figure S5. Reaction rate of cyclohexanol dehydration on (a) HMFI[5] and (b) HBEA at 150 °C as a function of as a function of the boundary distance (d_{b-b}) between two neighboring hydronium ions. The dash line represents the diameter of HMFI and HBEA micropores. The TOF of H-MFI in Figure S5a is reproduced with permission from Ref. 5. Copyright 2021, AAAS.

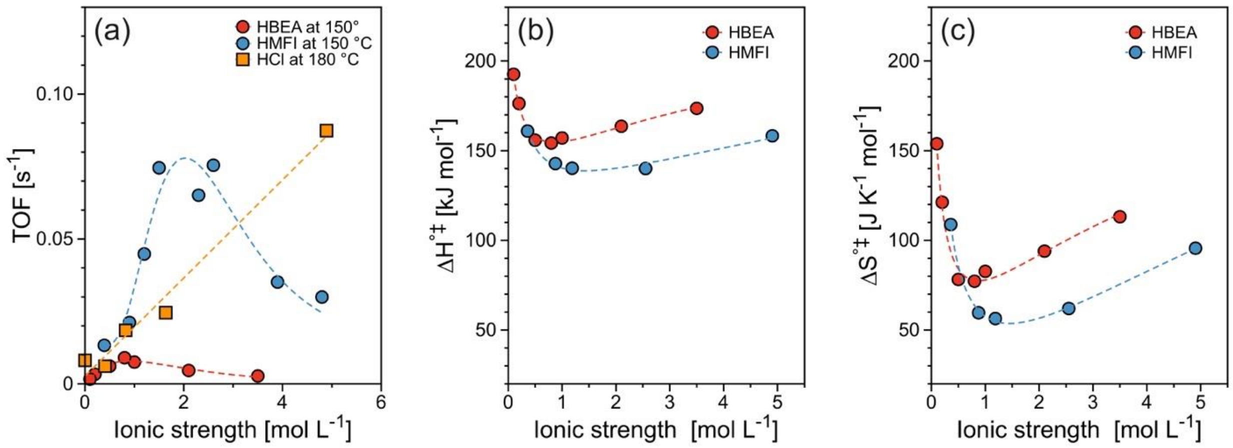

Figure S6. (a) Reaction rate of cyclohexanol dehydration at 150 °C, and (b) activation enthalpy ($\Delta H^{\circ\ddagger}$) and (c) activation entropy ($\Delta S^{\circ\ddagger}$) as a function of ionic strength. The TOF, $\Delta H^{\circ\ddagger}$, and $\Delta S^{\circ\ddagger}$ of H-MFI are reproduced with permission from Ref. 5. Copyright 2021, AAAS.

S. 4. Reference:

1. Prodinger, S.; Shi, H.; Eckstein, S.; Hu, J. Z.; Olarte, M. V.; Camaioni, D. M.; Derewinski, M. A.; Lercher, J. A., Stability of Zeolites in Aqueous Phase Reactions. Chem. Mater. 2017, 29 (17), 7255-7262.
2. Prodinger, S.; Shi, H.; Wang, H.; Derewinski, M. A.; Lercher, J. A., Impact of structural defects and hydronium ion concentration on the stability of zeolite BEA in aqueous phase. Appl. Catal., B 2018, 237, 996-1002.
3. Eckstein, S.; Hintermeier, P. H.; Zhao, R.; Baráth, E.; Shi, H.; Liu, Y.; Lercher, J. A., Influence of Hydronium Ions in Zeolites on Sorption. Angew. Chem. Int. Ed. 2019, 58 (11), 3450-3455.
4. Kim, S.; Jaegers, N. R.; Hu, W.; Hu, J. Z.; Chen, F.; Liu, Q.; Camaioni, D. M.; Derewinski, M. A.; Gutiérrez, O. Y.; Liu, Y.; Lercher, J. A., Impact of the Environment of BEA-Type Zeolites for Sorption of Water and Cyclohexanol. J. Phys. Chem. C 2023, 127 (48), 23390-23399.
5. Pfriem, N.; Hintermeier, P. H.; Eckstein, S.; Kim, S.; Liu, Q.; Shi, H.; Milakovic, L.; Liu, Y.; Haller, G. L.; Baráth, E., *et al.*, Role of the ionic environment in enhancing the activity of reacting molecules in zeolite pores. Science 2021, 372 (6545), 952.
